# Acknowledgement to Reviewers of *Genes* in 2018

**DOI:** 10.3390/genes10010048

**Published:** 2019-01-15

**Authors:** Genes Editorial Office

**Affiliations:** MDPI, St. Alban-Anlage 66, 4052 Basel, Switzerland

Rigorous peer-review is the corner-stone of high-quality academic publishing. The editorial team greatly appreciates the reviewers who contributed their knowledge and expertise to the journal’s editorial process over the past 12 months. In 2018, a total of 640 papers were published in the journal, with a median time to first decision of 23 days and a median time to publication of 45 days. The editors would like to express their sincere gratitude to the following reviewers for their cooperation and dedication in 2018:
Abdeltawab, NourtanLu, GangAbeywardana, TharindumalaLu, JingAckerman, MatthewLu, LinchaoAckermann, MaegenLu, YuanAdamowicz, SarahLucas, EmmaAdamus-Białek, WiolettaLucchesi, PaulaAddepalli, BalasubrahmanyamLucek, KayAdriaens, Michiel E.Ludwig, ArneAfonnikov, Dimitry ArkadievichLueck, John D.Agaphonov, MichaelLumniczky, KatalinAgarwal, SumitLuo, Ji-DungAguilera, FelipeLuo, MinkuiAhmed, Zubair M.Luque, RaulAjjampur, Sitara RaoLyle, RobertAkgül, BünyaminM’kacher, RadhiaAklilu, BehailuMa, LiangAlanis-Lobato, GregorioMa, QinAlekseyenko, Alexander V.Ma, XiaotuAleman, Ignacio TorresMa, YuAlemany, SilviaMaan, MartineAlexander, MathewMacholán, MilošAlhawarat, MohammadMaciukiewicz, MalgorzataAllan, Une ClasMacori, GuerrinoAllen, MichelleMacpherson, James N.Allen, RandyMacQueen, AliceAllender, CharlotteMacrander, JasonAllott, EmmaMadea, BurkhardAllred, Nicholette DanielleMaeda, YusukeAllweiss, LenaMafi Moghaddam, SamiraAlma D., Campos-ParraMafra, IsabelAlmeida, PedroMajor, FrançoisAlmeida, Rodrigo C.Majumder, MrinmoyeeAlmeida, VaniaMajumder, PrithaAlsford, SamMakhrov, Alexander A.Alshabib, EbtihalMakova, KaterynaAltmäe, SigneMakunin, Alexey I.Altman, Brian J.Malacrinò, AntoninoAlvarez-Ponce, DavidMalek, JoelAmenabar, MaximilianoMalerba, DonatoAn, XinminMalerba, GiovanniAndersen, CatherineMalone, JohnAndersen, EbbeMalpeli, GiorgioAndersen, Jeremy C.Manavalan, BalachandranAndrews-Cookson, MatiuMandal, LolitikaAndreyushkova, Daria A.Manickam, NandiniAngelini, CorradoMank, JudithAnquetil, VincentMansell, ThomasAntonov, IvanManzella, LiviaApplebaum, MarkMaraia, RichardArenas, MiguelMarchi, Fabio A.Arguelles, SandroMarco, FranciscoArmstrong, GaryMarigo, ValeriaArno, GavinMarin, TraciArrese-Igor, CesarMarla, SandeepArtemenko, YuliaMarshall, CharlaArtero, RubenMartínez, GermánArzalluz-Luque, AngelesMartínez-Cañamero, MagdalenaAscenzioni, FiorentinaMartínez-Espinosa, Rosa MaríaAsensi, VictorMartinez-Lostao, LuisAshikari, MotoyukiMartínez-Núñez, RocíoAssenza, GiovanniMarucci, LuciaAtaide, SandroMascitti, MarcoAtomi, HaruyukiMaskow, ThomasAttard, AgnèsMason-Suares, HeatherAttene, GiovannaMassa, AliciaAtzmon, GilMassade, LilianeAugustinos, AntoniosMasson-Boivin, CatherineAushev, VasilyMathews, David H.Aviran, SharonMatsuda, FumioAwan, ShakilMatta, JaimeAxtell, Michael J.Mattana, MonicaAyella, AllanMattarelli, PaolaAzarpajouh, SamanehMattei, BenedettaAzer, Samy A.Matthews, PhilippaBach, Duc-HiepMatucci Cerinic, MarcoBacusmo, Jo MarieMaurice, MadelonBai, YidongMauro, DanieleBains, PushpinderMc Auley, Mark T.Bak, SørenMc Manus, RossBaker, ScottMcClean, Phillip E.Bakkali, MohammedMcClung, RobertsonBalcerczyk, AnetaMcClure, PatrickBaldrich, PatriciaMcConnell, Erin M.Ban, YusukeMcCoy, RajivBanta, ScottMcDaneld, Tara G.Barash, DannyMcdonald, OliverBarbadilla, AntonioMcPhee, KevinBarbaro, BarbaraMeans, AnnaBarchenger, DerekMeech, RobynBar-Even, ArrenMeglic, VladimirBarquera, BlancaMehta, AngelaBarratt, MichaelMehta, VarshilBasler, KonradMeidanis, JoaoBathe, MarkMeier, JoanaBatista, Pedro J.Meier, U. ThomasBattista, JohnMeiklejohn, ColinBauer, StefanMelis, AnastasiosBauersachs, StefanMellado, EmiliaBechini, AlessioMeller, JaroslawBehrens, JürgenMellies, JayBell, ChristopherMelnyk, CharlesBellelli, RobertoMelo, Bruno F.Beloukas, ApostolosMelrose, JamesBen-David, YaacovMelters, DanielBender, KyleMenck, CarlosBen-Dov, Iddo Z.Mendelson, Tamra C.Benedik, MichaelMendes, JérômeBennett, James P.Menon, VipinBenvenuto, ChiaraMérai, ZsuzsannaBeran, Rudolf K. F.Mercati, FrancescoBergero, RobertaMereu, ElisabettaBergholz, TeresaMergeay, MaxBerthier, CelineMerrill, RichardBertoli, GloriaMesbah, NohaBertoni, BernardoMeškys, RolandasBertran, AndreMetcalf, Jessica L.Besnard, GuillaumeMeulenbelt, IngridBesteiro, SébastienMeyer, IrmtraudBevilacqua, PhilipMeyer, Ralph G.Bewick, AdamMichels, PaulBhat, NehaMidwinter, AnneBhattacharyya, DhananjayMiklas, PhilBhutoria, SavitaMilavetz, BarryBi, XinMiller, Joshua MBidarimath, MallikarjunMillet, OscarBidochka, Michael J.Milojevic, TetyanaBidwell, III, GeneMirzayanz, RazmikBielinsky, Anja-KatrinMisceo, DorianaBielska, EwaMistou, Michel-YvesBienert, Gerd P.Mitchison, Hannah M.Bills, Gerald F.Miura, SayakaBionaz, MassimoMneimneh, SaadBirchler, James A.Modesti, MauroBird, AmandaModrák, MartinBirgül-Iyison, NeclaMoehring, AmandaBirket, SusanMoellering, Douglas R.Bissonnette, NathalieMoin, MazaharBiswas, SantanuMoisa, SoniaBitard-Feidel, TristanMolassiotis, AthanassiosBitocchi, ElenaMolinier, JeanBitterman, Peter B.Montag, JudithBlackburn, GwylimMontañez, RaulBlackmon, HeathMonte, ElenaBlagojević, JelenaMontgelard, ClaudineBlanco, AntonioMoorhead, GregBlanz, JudithMora Garcia, SantiagoBlasi, BarbaraMoraes, Carlos T.Blaud, MagaliMoraes, Evandro MarsolaBodenreider, OlivierMoreira, Mônica F.Boehme, Karen A.Moreno, AdrianBohrer, Benjamin M.Moretti, MatteoBombarely, AurelianoMorita, MitsuhiroBombieri, CristinaMorou, EvangeliaBortoluzzi, StefaniaMosher, RebeccaBose, DebojitMoss, WalterBothner, BrianMotamedi, MoBottaro, SandroMou, HaiweiBoulling, ArnaudMoura, AlexandraBousquet-Antonelli, CecileMourier, TobiasBozzaro, SalvatoreMugnier, MonicaBracken, CameronMuleo, RosarioBradshaw, PatrickMuller, Claude P.Braganca, Judith M.Müller, JoachimBraliou, GeorgiaMuller, MichaelBramley, PeterMüller, VolkerBranco, SaraMuñoz Caffarel, MaríaBreckpot, JeroenMurcha, MonikaBremer, ErhardMusier-Forsyth, KarinBreschi, AlessandraMusolf, AnthonyBrestic, MarianMustafa, GhazalaBrevern, AlexandreMuszyński, ArturBrim, HassanMuthaiyan, ArunachalamBrinkmann, HennerMuthamilarasan, MehanathanBrito, Ana CatarinaMychaleckyj, JosyfBritt, Anne B.Nadal, AnnaBrobeil, AlexanderNadiminty, NagalakshmiBrook, Alan H.Nagata, YosukeBrosh, RobertNagoshi, EmiBrown, Anthony M.C.Nagwekar, JanhaviBrown, CarolynNagy, BelaBrown, JamesNaik, SurabhiBrumos, JavierNaim, ValeriaBrusslan, JudyNakagawa, TakuroBubici, GiovanniNakamura, MasatakaBudowle, BruceNakaoka, HirofumiBultas, JanNakashima, KazuoBuonaguro, LuigiNalluri, JosephBurger, MichaelNamekawa, Satoshi H.Bürger, ReinhardNanda, IndrajitBurgess, KayeNandi, SaikatBurman, Jonathon L.Nannas, NatalieBykhovskaya, YelenaNarayanan, AnandCaballero, DavidNatarajan, ChandrasekharCabral De Mello, DiogoNathan, MeenaCacabelos, RamónNaveed Yousaf, MuhammadCacchiarelli, DavideNawaz, Muhammad AmjadCáceres González, Francisco F. Núñez DeNaz, AliCadigan, KennethNdiaye, KalidouCai, DaguangNeckers, Len M.Calafell, FrancescNemkov, Travis G.Calcutt, MichaelNepal, MadhavCalera, José AntonioNettles, JamesCalvo, AnaNeubauer, PeterCammack, JonathanNeumann, JoachimCampbell, CoreyNi, MinCampbell, MichaelNianiou-Obeidat, IriniCampos, EricNibali, LuigiCanal, Clinton E.Nicodemus, KristinCao, ShaolongNiehrs, ChristofCao, ZheNielsen, Kåre LCapiati, Daniela AndreaNikel, Pablo IvanCappetta, MonicaNishimura, WataruCaragiulo, AnthonyNitulescu, George MihaiCarapelli, AntonioNjah, HasnaCardinale, MassimilianoNoel, EmilyCardoso, Taina FigueiredoNoordam, RaymondCarels, CarineNorder, HeleneCarey, MichelleNorton, PamelaCarillo, PetroniaNovakovic, BorisCarlier, AurélienNovikova, PolinaCarlomagno, TeresaNowicki, RomanCarmen Montero-Calasanz, Maria DelNunes, Márcio Roberto TexeiraCarmi, ShaiNussenzweig, AndreCarroll, ThomasNybom, HildeCarugo, DarioOberdoerffer, ShaliniCarvalho, Luiz Joaquim Castelo BrancoOchatt, SergioCasamassimi, AmeliaOchsenreiter, TorstenCasillas, SoniaO’Connor, RebeccaCastanera, RaúlOestergaard, Vibe HallundbækCastella, MariaOffer, StevenCava, ClaudiaOgami, KoichiCavaillé, JeromeOgunjirin, AdebowaleCavallini, AndreaOhno, KinjiCavallo, CarolaÖhrfelt, AnnikaCerchia, LauraOkleštková, JanaChabbert, MarieOksanen, HannaChae, HeeyoungOlatoye, MarcusChaix, RaphaëlleOlson, DanielChalivendra, Subbaiah C.Olsson, SannaChalker, DouglasOmar, AhmadChalla, GhanaOniga, Smaranda DafinaChan, Timothy A.Oosting, JanChandnani, RahulOpaliński, ŁukaszChang, SuhwanOppert, BrendaChapman, MarkOren, AharonCharkraborty, TrinadOrlov, YuriyCharlesworth, DeborahO’Rourke, JamieCharlier, CaroleOrton, Thomas J.Chassot, FrancieliOrzaez, DiegoChater, CasparOssowski, StephanChaudhry, BillOta, YosukeChauhan, AshviniOwens, GregoryChaves, RaquelPacifico, DanielaChen, GangPaek, JungwookChen, JinPage Utrilla, JesúsChen, LeileiPagel, MarkChen, TaipingPagliai, Fernando A.Chen, WeiPaijmans, Johanna L.A.Chen, XiangPal, ChandalChen, YunshunPalacios, José-ManuelCherif, EmiraPalacios-Gimenez, Octavio ManuelCheviron, Zachary A.Pallen, MarkChilds, Kevin L.Palma Sircili, MarceloChisenga, CarolinePalmer, AndrewChiurazzi, MaurizioPan, TaoCho, JungnamPanda, SayantanChojnowski, GrzegorzPandey, Avinash ChandraChopra, RatanPandey, RenuChoudhary, Kumari SonalPandolfini, TizianaChristiaens, OlivierPapageorgiou, Anastassios C.Christiansen, Ole BjarnePapageorgiou, AristotelisChung, DongjunPapantonopoulos, GeorgeChuva De Sousa Lopes, Susana MarinaParamithiotis, SpirosCiani, ElenaParcells, MarkCiccarelli, RenataParedez, AlexanderCiccolini, JosephParent, RomainCichy, KarenPark, Hee-SungCioffi, MarceloPark, Jong-InCitek, JindrichPark, Si JaeCiudad, CarlosParra, RobertoClark, GregParsch, JohnClark, SusanParsons, RichardClaverie-Martin, FelixPasantes, JuanClavijo McCormick, AndreaPaschou, PeristeraClemes Cardoso, DanonPaterson, Gavin K.Close, PierrePatil, AshwiniCmielewski, PatriciaPatrizi, BarbaraCoelho, CamilaPauchet, YannickCoelho, SusanaPaul, Narayan ChandraCohen, DavidPavankumar, TheethaColeman, JonathanPawelkowicz, MagdalenaCollado, Ricardo CaballeroPayot-Lacroix, SophieCollins, NickPedrolli, DanielleComander, JasonPeethambaran, BelaConesa, AnaPelizzola, MattiaConsonni, GabriellaPeltzer, AlexanderContreras-Moreira, BrunoPemov, AlexanderCooke Bailey, Jessica N.Pena, RomiCoombs, CatherinePenn, Linda Z.Cooper, Arthur J.L.Penzo, MariannaCorbel, VincentPepper, Alan E.Corbí, Angel L.Pepper, John W.Corchero, Jose LuisPeralta, DanielCornman, Robert (Scott)Peralta-Sánchez, Juan ManuelCorsinotti, AndreaPerchec Merien, Anne-MarieCosta, MarcioPerez De La Vega, MarcelinoCosta, Maria ManuelaPérez-Delgado, Carmen M.Costello, JamesPérez-Montaño, FranciscoCoulombe-Huntington, JasminPerez-Rodríguez, MarthaCoulon, StéphanePerišić, OgnjenCousminer, DianaPeriyasamy, PalsamyCoustham, VincentPerkins, Brian D.Cox, Timothy C.Perrin, NicolasCozzi, CristinaPervouchine, DmitriCrawford, LorinPetkevičiūtė, RomualdaCrespi, Bernard J.Petr, KotlíkCrespi, MartinPetrov, AntonCrosatti, CristinaPetruzzella, VittoriaCrozatier-Borde, MichelePfander, BorisCsaba, MáthéPfeifer, FelicitasCuevas Covarrubias, Sergio AlbertoPhillips, AndyCui, LongzhuPiantino Ferreira, Antonio JoséCui, RongfengPicard, ChristineCullen, PaulPico, AlexanderCullingham, CatherinePiferrer, FrancescCuming, Andrew C.Pino Angulo, AdrianCwalina, BeataPinto, MilenaDa Costa, MiltonPiontkivska, HelenD’Alessio, CeciliaPipan, BarbaraDamiani, DanielaPirooznia, MehdiD’Amico, DavidePirrello, JulienDandekar, Abhaya M.Pisabarro, Antonio G.Dannemann, MichaelPlatt, ThomasD’Argenio, ValeriaPleguezuelos, JuanDave, SandeepPócsi, IstvánDavenport, Emily R.Podini, DanieleDavidson, Nicholas O.Pollier, JacobDavis, BrianPopp, DennyDe Andrade, MarizaPostma, Alex V.De La Fuente-Nunez, CesarPotashkin, JudithDe La Haba, Rafael R.Potter, SallyDe La Herrán, RobertoPrat, SalomeDe La Iglesia, Daniel H.Pretorius, SakkieDe La Varga, HerminiaPrigione, ValeriaDe Martino, AndreaProbst, AlineDe Matos Simoes, RicardoProulx, GilbertDe Meyer, SofiePruneda-Paz, JoseDe Oliveira, CláudioPucciarelli, SandraDe Val, SarahPuigdomènech, PereDe Waard, VivianPulvirenti, AlfredoDeakin, JaninePyron, R. AlexanderDebelius, JustineQi, XinDebener, ThomasQian, ChengminDefossez, Pierre-AntoineQiao, LiangDehzangi, AbdollahQiu, DeyouDéjardin, JérômeQiu, HuanDeJesus, MichaelQiu, YiDel Galdo, FrancescoQiu, Yin-LongDel Real, GustavoQuerol, EnriqueDelany, Mary E.Quiñones, BeatrizDemarquoy, JeanRaden, MartindeMello, AndrewRadic, MarkoDemina, TatianaRagni, LauraDeng, XufangRagupathy, SubramanyamDeng, XumingRahmouni, KamalDennis, ElizabethRai, KrishanDensmore, Douglas M.Raikhy, GauravDepamphilis, MelvinRaina, MedhaDeram, AnnabelleRaj Kumar, Praveen KumarDerbyshire, MarkRaja, Huzefa A.Derreumaux, PhilippeRamachandran, VinoyDeryusheva, SvetlanaRamón G., Guevara-GonzálezDes Marteaux, LaurenRamos, António M.Desai, JigarRamos, Marco A.Desgagné-Penix, IsabelRampelli, SimoneDeshmukh, RupeshRapp, AlexanderDevaraj, AishwaryaRasteiro, RitaDevireddy, AmithRauch, JensDevisetty, Upendra K.Raudsepp, TerjeDey, Bijan K.Rausch, TobiasDhahbi, JosephRaval, ManmeetDhall, Rajinder KumarRavan, MaryamDhanasekaran, Saravana M.Ravinet, MarkDhar, Manoj KumarRawlence, NicDi Fagagna, Fabrizio D’addaRaynaud, CécileDi Giovanni, SimoneRebelo, SandraDi Rienzo, ValentinaReddy Janga, MadhusudanaDiCenzo, George CReddy, AnupamaDie, JoseReddy, Umesh K.Dievart, AnneRedell, MicheleDimitriadis, EvaReed, KentDing, LinRehrig, Erin M.Dluzen, DouglasReker, DanielDo, Duy NgocRemigi, PhilippeDobretsov, SergeyRenna, MassimilianoDodd, AntonyRevill, PeterDolgova, OlgaRevilla, EugenioDondini, LucaRewatee, GokhaleDong, XiaoRey, LuisDong, YiboReynoird, NicolasDonofrio, NicoleRhodes, Davina H.Donoghue, HelenRibas de Pouplana, LluísDopman, EricRidge, StephenDorn, MarcioRiffle, MichaelDou, XiaoguangRiganti Fulginei, FrancescoDoyle, JacquelineRigó, GáborDoyle, Jeff J.Robert Boisivon, HeleneDragomir, AndreiRobert, JacquesDrougard, AnneRoberts, Ian S.Du, PufengRobin, Arif Hasan KhanDu, WaRobinson, Gene E.Dubey, RaminRoca, XavierDuchaine, ThomasRocheleau, GhislainDuda, MałgorzataRockx, BarryĎudáková, ĽubicaRodrigue, NicolasDumont, BethRodrigues, Celia F.Dunning Hotopp, JulieRodrigues, Luis ReginaldoDutour, OlivierRodriguez Trelle, FranciscoDuwaerts, CarolineRodriguez, MonicaDyall-Smith, MikeRodríguez-Aguayo, CristianDziewit, LukaszRodriguez-Flores, JuanEaly, AlanRodríguez-Llorente, Ignacio DavidEddy, SeanRodriguez-Revenga, LaiaEduardoff, MayraRoe, AmandaEdwards, JeremyRogalski, MarceloEedunuri, Vijay KumarRogatis, Anna DeEhrenhofer-Murray, AnnRogozin, IgorEide, DavidRojas, Luis A.Eimes, JohnRolli, EleonoraEl Shamieh, SaidRomano, JuliaElameen, AbdelhameedRomby, PascaleElbasyoni, IbrahimRoncaglia, PaolaEl-Esawi, MohamedRoose, Mikeal L.El-Hodiri, HeithemRosa, Maria JoseEloe-Fadrosh, EmileyRosen, Benjamin D.Elofsson, ArneRosenberg, HeleneEne, Iuliana VeronicaRothbart, ScottEnguita, Francisco J.Roukos, VassilisEntelis, NinaRoux, Pierre-FrançoisEriksson, Maria E.Rovatsos, MichailErnst Seemann, StefanRowe, MelissahEscobar, Juan S.Rozhon, WilfriedEscudé, ChristopheRuan, JianhuaEspejo, JaimeRübsam, AnneEspeso, Eduardo A.Rubtsov, NikolaiEsteve-Romero, JosepRuggeri, PaoloEtheridge, RonaldRuiz-Argüeso, TomásEvanno, GuillaumeRuiz-Herrera, AuroraEvilia, CarynRuppel, SilkeEzaz, TariqRussell, StevenFabi, João PauloRussello, Prof. MichaelFabris, LindaRusso, Matteo A.Fagen, JennieRusso, PasqualeFaleiro, Maria L.Ryan, Daniel E.Fan, ChenguangRyan, MeganFan, JianglinSaarma, UrmasFang, David DSahuquillo Balbuena, ElviraFanning, SeamusSakai, HiromichiFantini, SebastianSakai, LynnFardeau, Marie-LaureSakuma, ShunFarré, MartaSalak-Johnson, JaneenFatih Akay, MehmetSalehin, MohammadFaux, NoelSalmon, PabloFeng, WenyiSalpini, RominaFeo, FrancescoSalvadori, SusannaFernández-Silva, IriaSampaio, Natalia G.Ferree, PatrickSamuelson, JohnFeulner, PhilineSanak, MarekFiliault, DanieleSanchez, SabrinaFilkov, VladimirSanchez-Elsner, TilmanFill, Taícia PachecoSandovici, IonelFiori Gradia, DanielaSaneyasu, TakaokiFischer, Hans-MartinSanglard, DominiqueFladung, MatthiasSantiago-Rodriguez, TashaFolco, Hernan DiegoSantini, AntonelloFonseca, NunoSantisteban, PilarForejt, JiriSantos, Ana PaulaForsberg, SimonSantos, Antonio I.Fotopoulos, VasileiosSarkar, MrinalFoulkes, NickSartini, BeckyFox, Edward M.Sato, FuyukiFozo, ElizabethSatoh, NoriyukiFradejas-Villar, NoeliaSatoh, ShigeruFrampton, GarrettSauer, KarinFranco, Carlos M.Sauvage, ChristopherFraser, LeylandScaletti Acciai, FedericaFreestone, PrimroseScarel-Caminaga, Raquel Mantuaneli Freire, JoaoScatena, RobertoFreude, KristineSchaaper, RoelFreudenreich, Catherine H.Schachtschneider, KyleFrey, LewisSchaefer, Jeremy S.Friis-Hansen, LennartSchaible, Hans-GeorgFu, FuyouScharf, BirgitFu, ShulanScheffler, Tracy L.Fu, Yong-BiScherberich, ArnaudFuchs, Thilo M.Schläppi, Michael R.Fueger, PatrickSchlick, TamarFujimoto, RyoSchmid, AmyFukunaga, KenjiSchmid, JanFukunaga, TsukasaSchmidt, Heiko A.Fusaki, NoemiSchmidt, MartinFusco, AlfredoSchmidt, MartinaFutschik, MatthiasSchmidts, MiriamGabler, NicholasSchmitz-Streit, Ruth A.Gaertig, JacekSchneeweiss, GeraldGaetano, CarloSchneider, AndréGagnaire, Pierre-AlexandreSchönherr, SebastianGahlaut, VijaySchrey, AaronGaj, ThomasSchröder, ReinhardGali, HimabinduSchug, ZacharyGalindo, AntonioSchulte-Hostedde, AlbrechtGalindo-González, LeonardoSchuster, Christopher F.Gall, JosephSchutte, BrianGalvez, JulioSchütz, MonicaGan, Ren-YouSchwartz, Robert E.Ganopoulos, IoannisSclavi, BiancaGao, LongScott, JacobGao, WeiSearle, JeremyGao, Wu-JunSebastian, AimyGao, YanzheSeddon, Philip J.Garagounis, ConstantineSedlazeck, FritzGarcia, SòniaSember, AlexandrGarcía-Estrada, CarlosSeo, Hak SooGarcía-García, FranciscoSergeant, KjellGarrett, RogerSerin-Harmanci, AkdesGase, KlausSerino, GiovannaGassman, Natalie R.Seroussi, EyalGatesy, John E.Servedio, MariaGauthier, BenoitSeshacharyulu, ParthasarathyGavenonis, JasonSghaier, HaïthamGavery, MackenzieShafee, ThomasGe, YubinShah, AjitGebiola, MarcoShao, QiangGehring, MarySharakhova, MariaGentile, FrancescoSharif, JafarGeorg, JensSharma, AlokGerdol, MarcoSharma, Amar DeepGerlich, WolframSharma, ManuGershenson, AnneShavrukov, YuriGeslain, RenaudShaw, Kerry L.Gharani, PedramShen, C.-K. JamesGhigna, ClaudiaShen, Huai-ShunGiannone, GregorySherif, Sherif M.Gigolashvili, TamaraShi, SuhuaGill, HarsharnShi, XiaobingGimenez, EstelaShih, DavidGioia, TaniaShim, RosalynGiovannangeli, CarineShinjo, Sueli Mieko ObaGiraud, TatianaShinozaki, KazuoGiuliani, AlessandroShort, KirstyGiulietti, MatteoShoshan, MariaGloag, RosŠiler, BranislavGoedert, JamesSilva, GabrielaGoel, PeeyushSilva, Sónia CarinaGoffart, SteffiSimeunovic, AndreaGolbeck, John H.Simmonds, MatthewGoldman, Gustavo H.Simon, IstvánGoldstein, RichardSimon, RichardGong, FadeSimonneau, ThierryGongora Castillo, ElsaSingh, AnuragGonzalez, Juan MiguelSingh, JugpreetGonzalez, VictorSingh, KashmirGonzalez-Barcala, Francisco JavierSingh, PriyankaGonzález-Martín, AntonioSingla, BhupeshGoold, Hugh DouglasSinha Ray, ShresthaGöringer, H. UlrichSinn, PatrickGostinčar, CeneSinnberg, TobiasGould, SvenSinoquet, ChristineGowher, HumairaSlatkin, Montgomery W.Gradia, DanielaSmadja, CaroleGraether, Steffen P.Smith, AlisonGramazio, PietroSofronov, GeorgyGraphodatsky, Alexander S.Sokoloski, KevinGrapputo, AlessandroSolé, XavierGrath, SonjaSoltani, AliGreen, CatherineSomarowthu, SrinivasGregory, JesseSon, Deok-sooGresshoff, Peter M.Sonawane, AbhijeetGriffiths, Cara A.Song, JianchengGrolmusz, VinceSong, JikuiGroot, AstridSong, JoeGroße-Brinkhaus, ChristineSong, QingxinGrueber, CatherineSoriano, Jose MiguelGrusak, Michael A.Sorokin, MaximGrzebelus, DariuszSoto-Reyes, ErnestoGuglielmetti, SimoneSoultos, Nikolaos D.Guidolin, Aline SartoiSoutourina, OlgaGulick, Patrick J.Sparvoli, FrancescaGumulya, YosephineSpella, MagdaGundamaraju, RohitSpellman, PaulGuo, YanSpielman, Stephanie J.Gupta, RadheySpooner, DavidGupta, VijayalaxmiSpoto, BelindaGurevich, Vsevolod V.Sprenger, HeikeGuyot, RomainSpyridopoulos, IoakimGuzhova, IrinaSquires, JamesGuzik, UrszulaSreerama, SubramanyaGyllenstrand, NiclasSrikulnath, KornsornGyöngyösi, MariannSrivastava, AkhilGyuhwa, ChungSrivastava, SarikaHaak, DavidStaats, Charley ChristianHaase, Astrid D.Stafford, PhilipHaddi, KhalidStafforst, ThorstenHague, SteveStawiski, Eric W.Hajirasouliha, ImanSteel, Laura F.Halazonetis, Thanos D.Steger, KlausHaldar, SumantoStehle, JörgHale, MatthewSteinbüchel, AlexanderHaley, BraddSteup, MartinHall, JamesStevens, Ann M.Hall, RandyStevens, RickHannenhalli, SridharSticchi, ElenaHannoufa, AbdelaliStiller, JosefinHansen, Anne-MarieStolzenberg, Danielle S.Hanson, JohannesStoneking, MarkHansson, BengtStornaiuolo, MarianoHaqq, AndreaStorz, GiselaHaque, InamulStricker, SigmarHarb, OmarStringer, Jessica M.Harbert, RobertStrode, ClareHardiman, GaryStröm, LenaHardouin, JulieSuarez-Vega, AroaHarkess, AlexSuau, PedroHarmanci, Arif OzgunSucgang, Richard S.Hart, Stephen L.Sudol, MariusHashimi, HassanSuhail, YasirHassan, Sherif T.S.Sullivan, BrianHasselmann, MartinSultan, FarazHauser, ElisabethSun, JieHausner, WinfriedSun, WeiHautamäki, VilleSun, YingjieHavgaard, JakobSun, ZhifuHawkins, JulieSundar, ReshmaHawley, ScottSung, PatrickHayes, Clair NelsonSung, SibumHazak, OraSupernat, AnnaHe, FeiSuryawanshi, VasantikaHe, Jia-QiangSusko, EdwardHe, Wei-QingSuzuki, YasutsuguHeckl, DirkSwerdlow, Russell H.Heggeset, Tonje Marita BjerkanSymkal, PetrHehlmann, RüdigerSymonova, RadkaHeilbronner, UrsSzabo, JuditHeitman, JosephSzuts, DavidHejtmancik, J. FieldingTadesse, WuletawHeller, Elizabeth A.Tai, WanboHelms, VolkhardTakagi, MasatoshiHendrix, DavidTakamatsu, DaisukeHéon, EliseTakezaki, NaokoHerbig, AlexanderTam, JosephHermoso, José Antonio MonrealTan, Roderick J.Herold, NikolasTan, Wen-HannHerrgard, MarkusTanabe, HideyukiHertweck, Kate L.Tardito, SaverioHerzberg, MartinTatarinova, TatianaHibbard, BruceTauber, EranHiggins, DeniceTauriello, DanieleHill, JanetTavío, María Del MarHillmann, FalkTedbury, PhilipHingorani, DinaTeixeira, LuisHinojosa, SilviaTeixeira, Miguel C.Hirbe, Angela C.Telenti, AmalioHirota, ToruTellier, MichaelHoang, Nam V.Temel, AslihanHofacker, IvoTemesvari, LeslyHoffmann, FedericoTempest, HelenHofman, CourtneyTemples, HeideHofreiter, MichaelTeng, ShaoleiHolalu, SrinidhiTeotia, PoojaHolland, Linda Z.Terracciano, DanielaHolland, Mitchell M.Terrazzino, SalvatoreHollenhorst, Peter C.Tessarz, PeterHolzmann, KlausTestolin, RaffaeleHoma, SherylTestoni, BarbaraHooda, JagmohanTetard-Jones, CatherineHooper, DanielTetreau, GuillaumeHornburg, DanielThalamuthu, AnbupalamHorvath, AneliaThapar, RoopaHoude, PeterTheis, Kevin R.Houldcroft, CharlotteTherkildsen, Nina OvergaardHowell, LynneThöny, BeatHu, BaoliThornton, Laura M.Hu, Yueh-ChiangTian, BinHuang, JianTimenetsky, JorgeHuang, LeiTimmis, JeremyHuang, WenTing, DavidHuang, ZixiaTittiger, ClausHuber, RobertTkavc, RokHulke, BrentToh, Tan BoonHumbeck, KlausTolias, Kimberley F.Humblot, PatriceTolwinski, NicholasHung, Kuo-HsiangTomanek, LarsHuq, EnamulTomar, DhanendraHusak, ZvenyslavaTong, HongningHuson, DanielTopalidou, EleniHuylmans, Ann KathrinTorchia, JonathonHvilsom, ChristinaTornesello, MariaHyun, Sang-HwanTorres, Adrian GabrielIbba, MichaelTorres, GabrielaIglesias, Alberto Á.Torres, JasonIlmer, MatthiasTost, JörgImai, KenichiroToulany, MahmoudInnocenti, NicolasTovo-Rodrigues, LucianaInoue, KentaroTrabucchi, MicheleInui, MasayukiTrammell, SamIrato, PaolaTrapp, JudithIsemura, TakehisaTremblay, KimberlyIsmail, Hanafy MahmoudTrojanowska, MariaIto, Ken-ichiTrost, Paolo-BernardoIvancic-Bace, IvanaTsang, StephenIvanov, Alexander V.Tu, MinIvanov, PavelTu, ThomasIvashchenko, DmitriyTuda, MidoriIvy, JamieTurner, DerekJabran, KhawarTurner, DouglasJackson, StephenTurner, JonathanJAFRA, SylwiaTyrka, MirosławJain, RachitTyson, GregoryJang, Tae-SooTzima, Aliki K.Janiak, AgnieszkaUchida, ShizukaJaramillo, Melba C.Uetz, PeterJarret, Robert L.Unchwaniwala, NuruddinJarvis, DavidUpadhyay, Rakesh K.Jauset, MiriamUptmoor, RalfJeffery, Constance J.Urano, DaisukeJeffrey Chen, ZengjianUrrestarazu, JorgeJemielity, JacekUsdin, KarenJena, Prasant KumarUygun, SahraJennings, LawrenceVaidya, AkhilJeremić, SanjaValdmanis, PaulJerez, Carlos A.Valenti, AnnaJetten, AntonValentini, AlessioJha, MithileshValenzuela, NicoleJin, YongfengValli, Adrian AlejandroJing, FuyuanVallone, PeterJohansson, PeterVan De Berg, WilmaJohnson, DanielVan Der Giezen, MarkJohnsson, MartinVan Der Linden, GerardJohnston, AndrewVan Haute, LindseyJones, Kathryn MarieVan Kessel, Julia C.Jones, MattVan Laar, TriciaJones, MichelleVan Loo, RobertJones, NeilVan Straalen, Nico M.Jones, NicolaVarallyay, EvaJones, Steven J.Varanini, ZenoJordá, LucíaVasaikar, SuhasJordan, Mark C.Vasconcelos, Daniel Fernando PereiraJose, MarcoVasconcelos, PedroJousselin, AmbreVashist, Yogesh KumarJovanovic, AleksandarVassilev, DimitarJugo, Begoña M.Vaux, FelixJulier, CécileVavitsas, KonstantinosJuneau, PhilippeVázquez-Santana, SoniaJungmann, RalfVear, FelicityJurečka, PetrVega, Francisco M.Jurkowska, Renata ZofiaVelegzhaninov, Ilya O.Kaakinen, MarikaVerde, GaetanoKale, AbhijitVerhaagen, JoostKalendar, RuslanVerheyen, EstherKaler, KaranVeronesi, FabioKamekura, MasahiroVersaw, WayneKamp, ChristelVerschoor, ChrisKaneda, MasahiroVicoso, BeatrizKang, CongBaoVieira, AlexandreKang, SeongVieira, Maria Lúcia CarneiroKang, Sung-TaegViennois, EmilieKantak, ChaitanyaVignal, AlainKappachery, SajeeshVihinen, MaunoKappes, FerdinandVillarino, GonzaloKaras, BogumilVinnedge, Lisa PrivetteKärenlampi, SirpaViola, IvanaKasarda, RadovanVitali, FrancescaKashatus, DavidViti, CarloKashina, AnnaVladimirov, VladimirKato, Takamitsu A.Vogt, ThomasKatoh, MasaruVOLCEANOV, AdrianKavas, MusaVolff, Jean-nicolasKay, GemmaVolleth, MarianneKeam, Simon P.Volschenk, HeinrichKędzierska, BarbaraVontas, JohnKeegan, LiamVriesekoop, FrankKehoe, PatrickVujosevic, MladenKeller, AdrianWaite, David WilliamKeller, Kate E.Wales, NathanKellner, RonnyWalker, Graham C.Kellner, StefanieWalker, Roy S. K.Kennedy, ScottWalkley, CarlKeung, Albert J.Wallner, BjörnKhan, IzharWalworth, Nancy C.Khetani, Salman R.Wan, DongshiKiefer, FriedemannWan, YueKierzkowski, DanielWang, Bang-AnKim, DaehwanWang, BoKim, HoyeunWang, En-DuoKim, Hyung SikWang, GregKim, Jin-WookWang, JiguangKim, Yong-IckWang, JunKim, Young-KookWang, KaiKimball, Rebecca T.Wang, LeiKinch, LisaWang, PeipeiKing, Jonathan L.Wang, QiKing, TuriWang, QiangKinsey, BrockWang, SiyunKlähn, StephanWang, SonghuKlaholz, Bruno PWang, XiaofeiKlein, HannahWang, XinKlocko, AmyWang, XuewenKnapp, MichaelWang, Yane-ShihKoblmüller, StephanWang, YangKocher, Thomas D.Wang, YumengKodedová, MarieWang, ZhengKoeleman, BobbyWani, Shabir HussainKoide, TieWaples, RobinKomissarov, AlekseyWarchałowska-Śliwa, ElżbietaKomiyama, TomoyoshiWard, Diane McVeyKommireddy, VasuWarner, RyanKomorowski, JanWashington, M. ToddKooij, Pepijn W.Watashi, KoichiKoonin, EugeneWattanapitayakul, Suvara K.Korac, AleksandraWaxman, JoshuaKoren, AmnonWay, JeffreyKorolev, KirillWaycott, MichelleKorostynski, MichalWebb, Ashley ElizabethKoskela, ElliWeber, Bernhard H.F.Kosova, KlaraWeber, FriedemannKostyn, KamilWei, Fu-WenKothe, UteWei, GanKotsiantis, SotirisWeigand, Julia E.Kozubowski, LukaszWeikard, RosemarieKraic, JanWein, NicolasKrasikova, AllaWeinberg, ZashaKratochvíl, LukášWeiss, JuliaKrejci, LumirWeiss, StevenKrieg, Adam J.Weiss, VivianKroese, DirkWellinger, Raymund J.Krupovic, MartWen, JiangqiKschischo, MaikWeng, Mao-LunKu, H. TeresaWessler, SusanKubo, YasutakaWest, ChristopherKuchipudi, SureshWeyrich, Laura SusanKuhlmann, MarkusWhyard, SteveKumar, BinodWilde, AnnegretKumar, DevenderWilke, ClausKumar, ManuWillert, KarlKumar, RajivWilliam, BartKumar, SudhirWilliams, GavinKumar, SunilWilliams, GrahamKumari, AshaWilliamson, JamesKumazawa, YoshinoriWilusz, JeffKunji, EdmundWinans, SteveKurjogi, MahanteshWindbergs, MaikeKushiro, TetsuoWitte, AngelaKusza, SzilviaWłoga, DorotaKuzmina, Maria L.Wolf, BethanyKwak, ShinWolf, LiorKwon, Yung-KeunWood, Craig CKyle, StuartWoolford, CarolKyriacou, CharalambosWu, FangxiangLa Rosa, MassimoWu, Pei-changLadomery, MichaelWu, RongxueLaenkholm, Ann-VibekeWu, SiLam, AnnaWurtele, HugoLambing, ChristopheXi, XuguangLanda, IñigoXia, YuchenLanfranchi, GerolamoXiao, JunLarkin, DenisXing, JunjiLarriba, EduardoXing, ZhuoLarsen, Lars AllanXu, JintaoLarson, Steven R.Xu, KeLashin, SergeyXu, LinLassalle, FlorentXu, WenyanLau, KinYadav, PankajLaurens, FrançoisYagi, MasafumiLavin, MattYamaguchi, YoshihiroLawrence, Jeanne B.Yamashita, AkiraLawrence, MartinYa-Ming, ChengLeanza, LuigiYampolsky, LevLebuhn, MichaelYancey, Paul H.Leclaire, SarahYanda, MuraliLeclercq, AlexandreYang, Chih-JenLedo, AnaYazar, SeyhanLee, Hwan YoungYe, XiongYingLehmann, UlrichYe, XiucaiLehner, StefanieYe, ZhujiaLei, ZhentienYeaman, SamuelLeibowitz, JulianYeo, Eugenia Li LingLeigh, Brittany A.Yoder, JeffreyLeiphrakpam, Premila D.Yoo, Joo-YeonLeitsch, DavidYoshikawa, HisaoLek, MonkolYoung, SeanLents, NathanYoussef, Noha H.Leo, Jack ChristopherYu, GangLeon Serrano, JavierYu, MeifangLesterlin, ChristianYuan, XueLetarov, AndreyYuen, LillyLeung, AlanYurchenko, Andrey A.Levanon, ErezZaballos, Miguel ALevens, David L.Zaccara, SaraLezza, Angela Maria SerenaZachariou, ZachariasLi, An-DongZaitlin, DavidLi, LingZambonelli, PaoloLi, MingZamyatnin, Andrey A., Jr.Li, XianranZappulla, DavidLi, YiZararsiz, GokmenLi, ZhenZaritsky, AriehLiang, Chun-MinZazzeroni, FrancescaLiao, ChenZdeněk, KubátLibault, MarcZeh, DavidLiebert, Uwe G.Zehbe, IngebergLiehr, ThomasZeilinger, SusanneLienkamp, SoerenZeitz, ChristinaLin, HaoZenoni, SaraLinacre, AdrianZenteno, RobertoLindor, Noralane M.Zhai, KuiLindsay, HowardZhang, BoLindtke, DorotheaZhang, ChangyiLing, JiqiangZhang, FanLing, MauriceZhang, HengyouLink, Christopher D.Zhang, HuanminLinnen, CatherineZhang, HuiLino, SaúlZhang, JinLiu, BingqiangZhang, PengLiu, Chao-linZhang, RuiyongLiu, ChunmingZhang, TingLiu, DongfeiZhang, WeiLiu, FanZhao, HuixianLiu, Guei-SheungZhbannikov, IlyaLiu, HongboZheng, QiLiu, MingchunZheng, XiaomingLiu, RengyunZhou, BangjunLiu, WeiZhou, JiaLobachev, KirillZhu, ZhouLoosli, FelixZima, JanLopez Campos, GuillermoZingg, Jean-MarcLopez-Camarillo, CesarZiora, ZytaLoPiero, AngelaŻok, TomaszLoreille, OdileZou, QuanLorenz, RonnyZucker, JeremyLou, PingZúñiga, GerardoLovering, RuthZuo, Ran


